# Well Recovered and More Creative? A Longitudinal Study on the Relationship Between Vacation and Creativity

**DOI:** 10.3389/fpsyg.2021.784844

**Published:** 2021-12-23

**Authors:** Christine J. Syrek, Jessica de Bloom, Dirk Lehr

**Affiliations:** ^1^Department of Business Psychology, Bonn Rhein-Sieg University of Applied Sciences, Rheinbach, Germany; ^2^Department of Psychology, Tampere University, Tampere, Finland; ^3^Department of HRM & OB, University of Groningen, Groningen, Netherlands; ^4^Department of Health Psychology and Applied Biological Psychology, Leuphana University of Lueneburg, Lueneburg, Germany

**Keywords:** changes in creativity, vacation, recovery, longitudinal, DRAMMA, holiday, detachment, mastery

## Abstract

The aim of this study was to investigate employees’ self-reported creativity before and after vacation and to examine the impact of recovery experiences (detachment, relaxation, mastery, meaning, autonomy, affiliation) on changes in creativity. The DRAMMA model of Newman et al. provides the theoretical background of our approach. Longitudinal data was assessed with four repeated measurements. The study encompassed data from 274 white-collar workers. Analyses showed that employees subjectively perceive their creativity to benefit not immediately after their vacation but 2 weeks later. Detachment was significantly related to lower creativity within persons, while mastery experiences explained differences in creativity between persons. This study provides a detailed picture of changes in creativity around vacations.

## Introduction

Vacationing has psycho-social benefits similar to, or even exceeding the benefits of leisure time spent over shorter periods, such as evenings or weekends at home (De Bloom et al., 2017). Travel experiences undertaken alone, as a couple, or as a family, may be conducive to self-reflection (Bosangit et al., 2015), encourage personal development (Noy, 2004), and strengthen social relationships (Shaw et al., 2008). Moreover, vacationing has a positive impact in terms of personal development, family experiences, learning and quality of life, perceptions of disadvantaged members of society, such as people with low income (McCabe and Johnson, 2013), disabilities, and physical or mental diseases (e.g., Hunter-Jones, 2004). Healthy employees can benefit from taking time off work and taking a vacation in terms of health complaints, burnout, and affective well-being (Fritz and Sonnentag, 2006; De Bloom et al., 2014a; Syrek et al., 2018). But how about creativity as a key aspect of job performance? Most employers seem to believe that vacations increase employee productivity, and the U.S. Travel Association reported that two out of three US executives expect that vacations increase creativity (Achor and Gielan, 2016). However, scientific evidence on vacations and creativity is scarce.

In Western societies, regular time off from work is considered an integral part of working life and vacations are an essential element of quality of life (Filep, 2012), not least because of the pressure to be “always on, never done,” recovery work during off-job time is increasingly difficult (Sonnentag et al., 2017). This has wide-ranging detrimental consequences for employees’ well-being and performance (e.g., Amelsvoort et al., 2003). Recovery describes the process of psychophysiological unwinding that offsets the strain process initiated by work demands (Sonnentag and Geurts, 2009), and vacations constitute the longest, consecutive period of respite from work (e.g., Eden, 2001).

Two systematic literature reviews underline the positive impact of vacations on employees’ health and well-being (De Bloom et al., 2009; Chen and Petrick, 2013), evident in indicators, such as fewer health complaints (of sleep impairment, etc.; Fritz and Sonnentag, 2006), enhanced life satisfaction (Gilbert and Abdullah, 2004), and reduced exhaustion (Westman and Etzion, 2001). Furthermore, two long-term epidemiological studies report that not taking vacations for a prolonged time is related to a higher risk of heart attacks, cardiovascular disease, and even premature coronary death (Eaker et al., 1992; Gump and Matthews, 2000).

The link between vacationing and job performance, however, has received much less research attention. This is surprising, because diary studies on recovery episodes of shorter durations, like evening hours or weekends, indicate that employees who start their workday feeling mentally and physically refreshed report higher task performance (Binnewies et al., 2008). A recent study by Miyakawa et al. (2019) showed that travel frequency was related to an increase in generic skills (e.g., effective communication) and in the long run with employees´ monthly salary.

This study focuses on the impact of vacation, a prolonged episode of recovery from work, as a potential predictor of creativity as a key aspect of employees’ job performance. Researchers see creativity as one of the most complex and advanced achievements of which a person is capable (Taylor, 1988). Creativity can be described as “the production of novel and useful ideas by an individual or small group of individuals working together” (Amabile, 1988, p. 126). Employees’ creativity is an important potential competitive advantage for companies (Amabile, 1988). Therefore, organizations pursue the goal of promoting employees’ creativity to keep up with change processes and accelerated technological development (Volmer et al., 2019).

Numerous theories and studies aim to identify personal and organizational predictors of employees’ creativity at work (see Hammond et al., 2011, for a meta-analytical review). Yet the association between recovery and creativity has received scant research attention, although several theories and studies imply that there may be a connection between recovery and the generation of creative solutions (Fink et al., 2010; Kühn et al., 2014). A first study by De Bloom et al. (2014b) showed that employees’ cognitive flexibility increased after vacation.

In the present study, theories and methodological approaches are derived from tourism research, leisure sciences, and psychology aiming to connect research fields and build bridges between hitherto only loosely connected areas of expertise. Our focus is to further our understanding of the link between vacationing and creativity. To scrutinize the relationship between recovery and changes in creativity, we follow Newman et al. (2014) model to differentiate between recovery experiences (detachment, relaxation, autonomy, mastery, meaning, affiliation). Specifically, we examine employees’ self-reported work-related creativity. We apply a longitudinal research design with repeated measurements across a vacation period. Multilevel modeling is used to analyze the association between recovery experiences and creativity within persons as well as between persons. In general terms, our study aims to provide a detailed picture of the changes in creativity relating to vacationing.

## Mechanisms Connecting Vacations and Creativity

Why might vacations be potentially linked to creativity? Unstructured free time is assumed to alleviate people’s sense of urgency and encourage reflective thinking and incubation, which are crucial to creativity (Elsbach and Hargadon, 2006). Vacation is the longest consecutive period of leisure time and a prime opportunity to provide people with mental space for reflection, building the base for creative thoughts. Moreover, vacations provide people with beneficial experiences known to reduce work stress (Sonnentag and Fritz, 2007). According to Newman et al. (2014), these experiences can be grouped into six categories, summarized in the so-called DRAMMA model, which explains that subjective well-being is enhanced by leisure through satisfaction of the following psychological needs: detachment-relaxation, autonomy, mastery, meaning, and affiliation. Detachment and relaxation serve the need to refill resources depleted during work, while autonomy, mastery, meaning, and affiliation primarily fulfil the need to acquire new resources and experience pleasure.

### Detachment, Relaxation, and Creativity

*Detachment* focuses on mental unwinding and on gaining inner distance from work, (Sonnentag and Fritz, 2007) and shows the most consistent picture regarding its beneficial impact. Vacations represent a break from daily routine and hassle, thereby providing a good opportunity to mentally disengage from work. Schon (1983, as cited in Elsbach and Hargadon, 2006) argues that creativity stems from oscillating between involvement and detachment, underlining the importance of intermittent detachment from work tasks. Furthermore, detachment is a key mechanism for relieving stress (Sonnentag et al., 2017). Stress relief in turn improves creativity, particularly cognitive flexibility (De Bloom et al., 2013). More directly, Smith and Blankenship (1989) postulated the “forgetting-fixation” hypothesis, which states that turning away from preoccupation with an unsolvable problem makes it possible to forget wrong solutions, increasing the chances of finding a new creative solution. Thus, mental disengagement from work-related problems during vacation corresponds to the incubation phase which precedes creative thoughts as described by Sio and Ormerod (2009) and Ritter and Dijksterhuis (2014).

*Relaxation* is characterized by physiological unwinding and low levels of activation (Sonnentag and Fritz, 2007). The association between relaxation and creativity finds support in a study by Grabner et al. (2007), who found that increased alpha wave activity, especially in the right hemisphere of the brain, was associated with increased levels of originality in a creativity test. Further support for the association between relaxation and creativity can be found in the Default Mode Network (DMN), which consists of a set of different brain regions that are active when the brain seems to be resting (Callard and Margulies, 2014). The DMN allows us introspections and to experience daydreams, and is important for mental imagination and creativity. Kühn et al. (2014) found a positive association between creative performance (operationalized with the Alternative Uses Task) and the grey matter volume of the DMN, which implies that the DMN plays a crucial role in generating creative ideas. Employees often associate vacations with activities enhancing relaxation (De Bloom et al., 2013). Thus, relaxation during vacation is expected to be related to increased creativity.

### Autonomy, Mastery, Meaning, Affiliation, and Creativity

To understand the relationship between the four DRAMMA experiences autonomy, mastery, meaning, and affiliation and creativity, the connection between positive affect and creativity is vital. Vacations are a highly influential means to enhance employees’ positive affect and related constructs, such as happiness, well-being, and satisfaction (e.g., Kühnel and Sonnentag, 2011; Chen et al., 2013). Thus, in line with De Bloom et al. (2014b) and building on Frederickson’s broaden-and-build theory (2002), vacations are likely to increase employees’ creativity through the experience of positive affect during vacation. Positive affect is assumed to widen people’s attentional focus and repertoire of actions and cognitions. It encourages people to explore and discover, two important elements in the creative process (Fredrickson, 2002). Autonomy, mastery, meaning, and affiliation promote positive affect. Besides this general link between these DRAMMA experiences and creativity, we will describe in the following pages possible relationships based on further theoretical reasons and indirect evidence.

*Autonomy* represents a basic human need characterized by the desire to decide one’s own course of action (Deci and Ryan, 2000). Autonomy has been associated with higher creativity and cognitive flexibility in several experiments by Amabile and is a key component of Amabile et al. (1996) conceptualization of a climate for creativity. Similarly, several intervention studies also found support for the relationship between support for autonomy and creativity (Greenberg, 1992; McLachlan and Hagger, 2010). According to Deci and Ryan (1987), autonomous activities are regulated more flexibly with less tension and a more positive tone; they entail an inner endorsement of one’s action. Thus, they assume that “creativity, it seems, is fostered by events and contexts that support autonomy” (Deci and Ryan, 1987, p. 1029). As people typically choose their vacation destination and their activities with a high degree of autonomy, according to their options and preferences, one could speculate that autonomy experienced during vacation is related to higher creativity.

*Mastery* describes the experience of success or achievement which emerges from facing challenging situations. The need to extend physical and psychological skills is a basic human need (Deci and Ryan, 2000); mastery occurs in situations with a perfect balance between skill and challenge, followed by a sense of accomplishment. Vacations offer opportunities to broaden one’s horizon and leave one’s comfort zone (e.g., Noy, 2004). Such mastery experiences can result in higher self-efficacy (Binnewies et al., 2010). Similar to the concept of mastery, Stebbins (2005) argues that “serious leisure” (the leisure pursuit of an amateur requiring high levels of knowledge, skill, and experience) enhances self-actualization, self-enrichment, and a renewal of the self. Yau (1991) argues that a positive self-image is key to creativity. Empirical evidence stems from studies showing that self-esteem and self-efficacy are related to higher creativity (Goldsmith and Matherly, 1988). Further, diversifying experiences (such as mastery experiences) enhance creativity by broadening people’s knowledge pool and increasing processing depth. Ritter et al. (2012) found that involvement in an unusual event was associated with an increase in cognitive flexibility; studies in the field of multicultural experiences align with this finding (e.g., Cheng et al., 2011). Based on this indirect empirical evidence and theoretical assumptions, we expect mastery experiences during vacation to increase creativity.

*Meaning* characterizes the need to achieve a sense of purpose in life and doing something useful (Stebbins, 2005), which promotes peace of mind, self-worth growth, and social engagement (Newman et al., 2014). Vacations can provide meaningful experiences, such as broadening one’s horizons, by learning about new cultures or historical events and enhancing one’s self-reflection by meditating and calming down. Stephens and Carmeli (2017) argue that meaningfulness enables people to thrive – to experience aliveness and learning (Spreitzer et al., 2005) – which is essential for creativity. Similarly, meaningfulness in the workplace has been associated with enhanced creativity, a link which has been explained by the association between meaningfulness and positive psychological states (Cohen-Meitar et al., 2009). Thus, based on the literature, we expect a positive relationship between meaningfulness experienced during vacation and creativity.

*Affiliation* refers to the need to feel connected to others, to be loved and cared for, and to love and care (Baumeister and Leary, 1995). Several theories regard affiliation as a basic human need (e.g., Deci and Ryan, 2000). Affiliation is also a key trigger for positive affect (Newman et al., 2014). Social leisure activities enhance affiliation and promote positive emotions (Brajša-Žganec et al., 2011). The association between affect and creativity has often been investigated, consistently confirming the beneficial nature of positive affect for creative behaviors (e.g., Amabile et al., 2005). The experience of positive affect enhances cognitive flexibility, broadens access to a wider range of perspectives, and thus triggers the exploration and the generation of new ideas, which is corroborated by numerous studies (e.g., Ashby et al., 2002). Vacations are an excellent means to fulfil the need for affiliation, as studies show that vacations create important family memories and strengthen relationships to family members and partners (Lehto et al., 2009). Therefore, based on indirect empirical evidence, affiliation experienced during vacation is expected to enhance creativity.

Summing up, several theories and earlier findings from empirical studies support the assumption that vacationers may benefit from the fulfillment of the six DRAMMA experiences in terms of creativity. Yet empirical research is scarce on how the experience of DRAMMA during vacation relates to creativity after vacation (compared to creativity before vacation). This study aims to bridge this research gap. More specifically, we hypothesize:

H1: Employees’ creativity is higher after vacation than before vacation within persons.

H2: Recovery experiences (detachment, relaxation, mastery, meaning, autonomy, affiliation) predict changes in employees’ creativity within and between persons.

## Materials and Methods

### Procedure and Design

The participants were 274 German employees working in various industries. Information regarding confidentiality, as well as the voluntary nature of participation, was posted on a website online along with the announcement of the study. Of the 339 employees who had provided their email information, confirmed their interest in participating and reported their individual vacation period, 274 responded to the questionnaires. Two weeks prior to their vacations, we sent all registered employees a link with a baseline survey assessing work and background characteristics. Employees then responded to brief online questionnaires on five measurement points: 2 weeks before vacation, the last day of work before their vacation, during their vacation (=middle of the holiday), on their first day back at work, and 2 weeks after the vacation. While recovery experiences were measured during vacation, self-reported work-related creativity was not measured during vacation. Employees were offered the option of using a smartphone application (that accompanied them) before, during, and after vacation with proposals for tasks to increase recovery.

### Sample

The data set included 274 employees and 752 measurements, indicating a completion rate of 25% for self-reported work-related creativity. Respondents in the dataset respond to at least three measurement points. Of the sample, 74% were female. Employees were between 18 and 67-year-old (*M* = 40.20, *SD* = 10.80). Most employees were married or cohabiting (78%), and 39% had children. Average tenure was 9 years (*SD* = 8.97), ranging from less than 1–40 years. Most employees worked full time (73%). The majority of participants had a college degree (58%), followed by intermediate level (21%) and high school levels (21%) of education.

### Vacation Information

On their last day at work, we asked participants several details about their upcoming vacation to get a better picture. Almost half (48%) planed wellness activities, 36% going to the beach, 33% reported having planned sporting activities (e.g., hiking, sailing, and cycling), 31% spending time in natural surroundings, 31% staying at home during their vacation (including day trips), 20% visits to relatives, 10% planned a self-organized round trip, 13% a city trip, 11% cultural activities. Of the sample, 58% planned to travel in Europe (outside Germany), 17% to travel outside Europe, 13% in Germany, and 12% to stay at home.

Concerning travel companions, 65% reported going on vacation with their partner, 24% with their children, 22% with family, 14% with friends, 3% alone, and 1% with colleagues (multiple answers possible). On their first day back at work, participants on average felt that their vacation was fairly good (*M* = 7.19, *SD* = 1.63 on a scale from 1 = this vacation was a flop to 10 = this vacation was the vacation of my life).

We explored if creativity and recovery experiences differed depending on vacation destination and content of vacation. While differences in creativity were not associated with destination or content, employees experienced lower detachment when staying at home during their vacation. Employees who were going to the beach reported higher relaxation, city trips were related to lower relaxation. Sporting activities were associated with higher autonomy. Employees vacationing with partner and children reported higher affiliation.

### Measures

We assessed creativity with a self-reported measure. *Self-reported work-related creativity* was assessed at four measurement points: 2 weeks prior to the vacation, on the last day at work, on the first day back at work, and 2 weeks after the vacation with three items adapted from George and Zhou (2001): “My head is full of creative and innovative ideas that are related to my work,” “My head was full of creative solutions to work-related problems,” “My head was full of ideas to solve work tasks in a new way.” Participants responded on a five-point Likert scale (1 = totally disagree, 5 = totally agree). Mean Cronbach’s alpha was 0.90.

In addition, we measured *creative performance* with the Alternative Uses Task, however, due to the lack of counterbalancing the written prompts before and after vacation, results are not reported.

*Recovery experiences* were assessed at five measurement points: 2 weeks prior to vacation, on the last day at work, during the vacation, and on the first day back at work, as well as 2 weeks after returning from vacation. The questionnaire used to assess recovery experiences builds on and combines existing items to cover all dimensions of the DRAMMA model, referring to the participant’s leisure time. In this questionnaire, detachment is measured with three items adapted from the well-validated recovery experience questionnaire (Sonnentag and Fritz, 2007) and the rumination scale (Mohr et al., 2006). Relaxation and mastery are measured using the well-validated recovery experience questionnaire with three items each (Sonnentag and Fritz, 2007). To measure meaning, three items from the “job diagnostic survey” (Hackman and Oldham, 1974) were reformulated to apply to leisure time. Autonomy and affiliation are each measured with three items adapted from the “Basic Need Satisfaction in General Scale” (Johnston and Finney, 2010). Participants responded on a five-point Likert scale (1 = totally disagree, 5 = totally agree). The DRAMMA model has been validated with regard to vacation experiences and well-being by Kujanpää et al. (2020). Mean Cronbach’s alpha was 0.94 for detachment, 0.95 for relaxation, 0.83 for autonomy, 0.94 for mastery, 0.92 for meaning and 0.93 for affiliation. All items are provided in an online supplementary.^1^

We tested our hypotheses with app usage as a control variable in order to control its effect and assess the changes in creativity before and after vacation. As app usage did not change the pattern of the results and did not explain variance in creativity, this control variable is not modeled in the final analyses.

### Analytical Strategy

To analyze changes in recovery experiences and self-reported work-related creativity, we used multilevel modeling techniques to account for the systematic and chronological structure of time, as well as the nonindependence of the data. We followed Bliese and Ployhart (2002) five-step approach and estimated multilevel models in R, using the NLME library. Restricted maximum likelihood was used for estimation. In the first step, we determined the strength of nonindependence and estimated ICC(1,k; Koo and Li, 2016) to examine the amount of variance in individual ratings in DRAMMA experiences and in self-reported creativity that is due to interindividual differences and analyze if there is substantial variance within persons across measurement points. In the second step, we modeled linear as well as quadratic and cubic trends of time and followed Bliese and Ployhart (2002) approach by converting time into power polynomials. In the third step, we tested if allowing the slope of time to randomly vary fits the data better. In the fourth step, we assessed the error structure of the model by including (a) an autoregressive structure with serial correlations and (b) incorporating heterogeneity in the error structures. In the fifth step, we analyzed the relationship between recovery experiences and creativity. The R script is provided in an online supplementary. Multilevel analyses make it possible to model between-person effects and within-person effects at the same time. We therefore investigated the intraindividual relationships of recovery experiences and creativity as well as interindividual relationships, that is, we examined whether, for points in time at which employees’ experienced more recovery than at other points in time, their creativity was higher (intraindividual level), and also whether employees experiencing more recovery than other employees reported more creativity (interindividual level). We followed Raudenbush and Bryk (2002) approach and included recovery experiences as predictors (person-mean centered, depicting within-person variance) and their aggregates (grand-mean centered person-means, capturing the overall level of recovery experiences), thus decomposing the effect into within- and between-person components. We included all six recovery experiences as predictors in one model, as confirmatory factor analyses by Kujanpää et al. (2020) showed that the six experiences can be differentiated even though they intercorrelate.

## Results

Table 1 presents the zero-order correlations between study variables.

**Table 1:** Means, SD, and correlations between study variables.

	*M*	*SD*	1	2	3	4	5	6	7	8	9
1 Gender^a^	1.26	0.44									
2 Age	31.74	10.81	0.23^**^								
3 Creativity	2.62	0.82	0.16^**^	0.12^*^		−0.05	−0.02	0.00	0.05	0.04	0.00
4 Detachment	2.97	0.78	−0.05	0.00	0.15^*^		0.78^***^	0.65^***^	0.32^***^	0.37^***^	0.47^***^
5 Relaxation	2.87	0.77	0.01	0.04	0.24^**^	0.56^**^		0.76^***^	0.38^***^	0.43^***^	0.56^***^
6 Autonomy	3.26	0.69	−0.01	−0.04	0.18^**^	0.51^**^	0.69^**^		0.44^***^	0.44^***^	0.57^***^
7 Mastery	3.00	0.85	−0.02	−0.02	0.28^**^	0.17^**^	0.33^**^	0.23^**^		0.62^***^	0.33^***^
8 Meaning	3.17	0.86	−0.08	−0.10	0.19^**^	0.31^**^	0.45^**^	0.33^**^	0.70^**^		0.40^***^
9 Affiliation	3.97	0.57	−0.07	−0.06	0.15^**^	0.35^**^	0.35^**^	0.45^**^	0.16^**^	0.33^**^	

*For the sake of economy, correlations below the diagonal are person-level correlations, self-reported work-related creativity and recovery experiences are averaged across all measurement points (N = 274), correlations above the diagonal are week-level correlations (N = 752)*.

a *Male = 1, female = 2*.

*** *p < 0.001*;

** *p < 0.01*;

* *p < 0.05*.

### Development of Recovery Experiences Across Time

**Figure 1:**
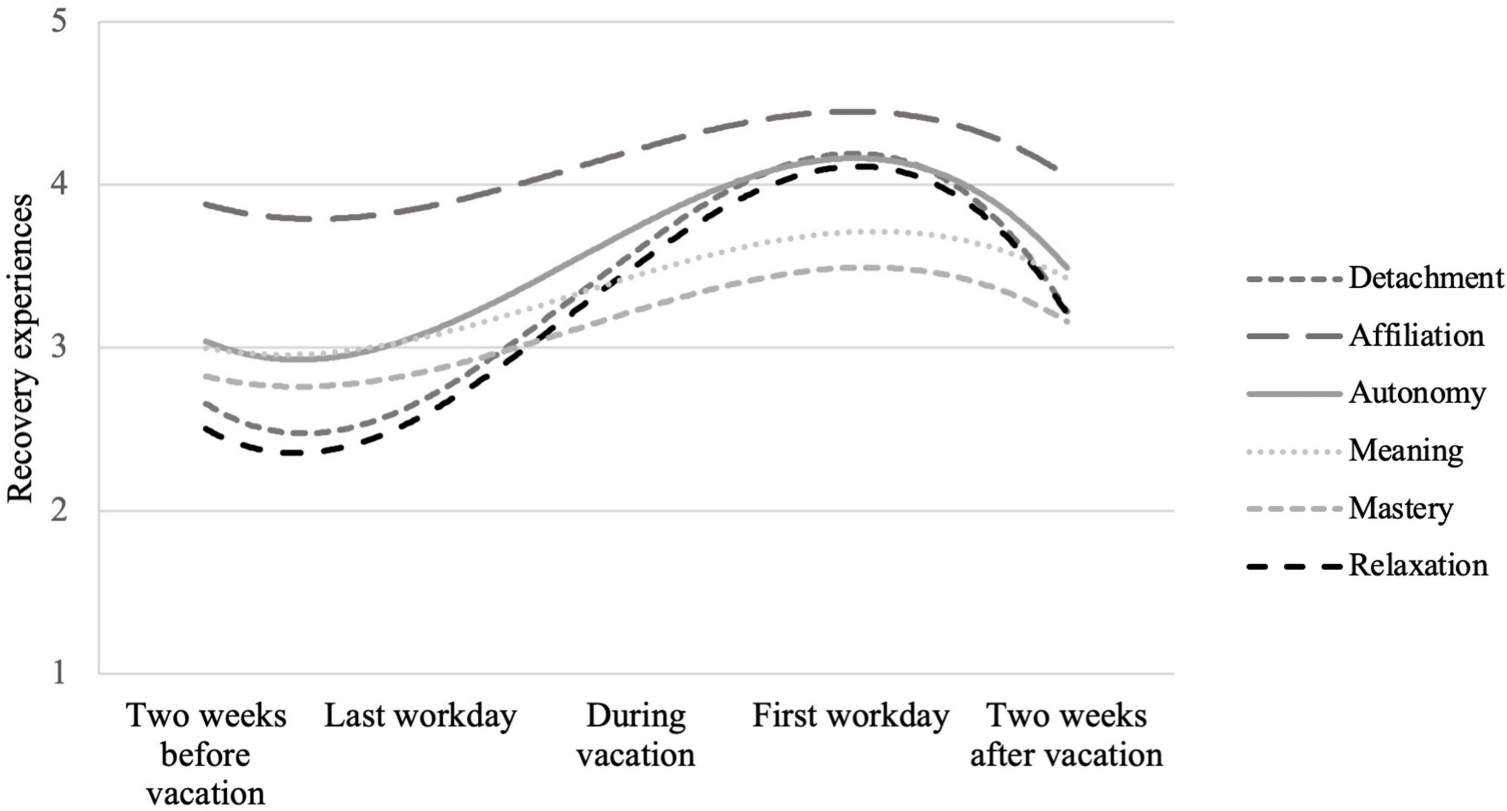
Estimated trajectories of DRAMMA experiences across time on a five-point Likert scale.

ICC(1,k) for the DRAMMA experiences ranged between 0.19 and 0.42, showing a considerable amount of within-person variance. We modeled time and analyzed linear, quadratic as well as cubic time trends by converting time into power polynomials. The results show that all three time trends (positive linear trend, negative quadratic trend, negative cubic trend) were significant for each DRAMMA dimension, indicating that recovery experiences decreased from 2 weeks prior to the vacation to the last day at work before the vacation, increased during the vacation, and again slightly decreased 2 weeks after the vacation (Tables 2 and 3). Figure 1 shows the average trajectory of all DRAMMA experiences across all participants. Mean scores are provided in an online supplementary.

**Table 2:** Growth model analysis for detachment, relaxation, and autonomy.

	Detachment			Relaxation			Autonomy		
	*γ*	*SE*	*t*	*γ*	*SE*	*t*	*γ*	*SE*	*t*
Intercept	3.21	0.04	74.10^***^	3.11	0.04	72.71^***^	3.45	0.04	94.05^***^
Time linear	12.62	0.77	16.31^***^	13.37	0.76	17.65^***^	8.98	0.70	12.82^***^
Time quadratic	−7.61	0.75	−10.10^***^	−7.27	0.74	−9.85^***^	−5.40	0.68	−7.90^***^
Time cubic	−11.11	0.74	−14.93^***^	−1.71	0.73	−14.70^***^	−7.77	0.68	−11.48^***^
L2 intercept variance (SE)	0.33 (0.57)			0.32 (0.57)			0.22 (0.46)		
L1 intercept variance (SE)	0.53 (0.73)			0.51 (0.72)			0.44 (0.67)		
BIC	2407.39			2371.38			2194.34		
AIC	2,378,35			2342.35			2165.30		
-2LL	2366.35			2330.35			2153.30		

*N = 274, Obs = 752, measurement points: Five (2 weeks before vacation, last workday, during vacation, first day of work, 2 weeks after vacation)*.

*** *p < 0.001*.

**Table 3:** Growth model analysis for mastery, meaning, and affiliation.

	Mastery			Meaning			Affiliation		
	*γ*	*SE*	*t*	*γ*	*SE*	*t*	*γ*	*SE*	*t*
Intercept	3.09	0.05	65.41^***^	3.28	0.05	68.95^***^	4.07	0.03	126.28^***^
Time linear	6.00	0.80	7.54^***^	6.50	0.77	8.45^***^	4.39	0.55	7.93^***^
Time quadratic	−2.93	0.77	−3.78^**^	−2.77	0.75	−3.70^**^	−3.00	0.54	−5.57^***^
Time cubic	−4.20	0.76	−5.51^***^	−3.97	0.74	−5.39^***^	−4.53	0.53	−8.53^***^
L2 intercept variance (SE)	0.41 (0.64)			0.43 (0.66)			0.19 (0.43)		
L1 intercept variance (SE)	0.56 (0.74)			0.52 (0.72)			0.27 (0.52)		
BIC	2483.14			2402.99			1764.57		
AIC	2454.10			2432.02			1793.60		
-2LL	2442.10			2390.99			1752.57		

*N = 274, Obs = 752, measurement points: five (2 weeks before vacation, last workday, during vacation, first day of work, 2 weeks after vacation)*.

*** *p < 0.001*;

** *p < 0.01*.

### Changes in Self-Reported Work-Related Creativity Across Time

Multilevel modeling showed that ICC(1,k) for self-reported work-related creativity was 0.42, indicating that almost half of the variance in creativity stemmed from differences between persons, and that there was also substantial variance within persons across time. The linear time trend (*γ* = 0.41, *t* = 0.53, *p* = 0.60) was not significant while the quadratic time trend (*γ* = 2.67, *t* = 3.50, *p* < 0.001) was significant (model 1, Table 4), indicating that employees subjectively perceived their creativity to be lower immediately after the vacation but reported benefiting 2 weeks after the vacation in terms of creativity – suggesting that vacation experiences needed time to unfold and become perceptible. The slope of time did not randomly vary. A model including autocorrelation and incorporating heterogeneity in the error structures did not fit the data better.

**Table 4:** Multilevel analysis predicting self-reported work-related creativity.

	*Model 1*			*Model 2*			*Model 3*		
	*Est*	*SE*	*t*	*Est*	*SE*	*t*	*Est*	*SE*	*t*
Intercept	0.62	0.05	54.29^***^	0.61	0.05	56.71^***^	2.60	0.05	56.54^***^
Time linear	0.41	0.79	0.53	0.14	0.88	0.16	−0.02	0.88	−0.02
Time quadratic	0.67	0.76	3.50^***^	0.61	0.76	3.42^***^	2.49	0.76	3.27^***^
Detachment within				−0.13	0.05	−2.39^*^	−0.13	0.05	−2.49^*^
Relaxation within				0.03	0.07	0.52	0.04	0.07	0.55
Autonomy within				0.02	0.07	0.35	0.03	0.07	0.42
Mastery within				0.08	0.05	1.46	0.09	0.05	1.60
Meaning within				0.04	0.06	0.69	0.04	0.06	0.61
Affiliation within				0.01	0.07	0.13	0.01	0.07	0.08
Detachment between				0.04	0.08	0.56	0.04	0.08	0.54
Relaxation between				0.18	0.10	1.81	0.17	0.10	1.79
Autonomy between				0.01	0.10	0.13	0.01	0.10	0.11
Mastery between				0.25	0.08	3.12^**^	0.26	0.08	3.23^**^
Meaning between				−0.09	0.09	−1.05	−0.09	0.09	−1.09
Affiliation between				0.09	0.10	0.92	0.10	0.10	1.04
time linear x Mastery							1.98	1.03	1.91
Time quadratic x Mastery							2.03	1.00	2.04^*^
L1 slope var. (SE)	0.54 (0.74)			0.53 (0.73)			0.53 (0.73)		
L2 intercept variance (SE)	0.40 (0.64)			0.35 (0.59)			0.35 (0.59)		
ΔPseudo *R*^2^ L1				0.01			0.00		
ΔPseudo *R*^2^ L2				0.14			0.00		
BIC	1998.43			2077.14			2092.10		
AIC	1975.33			1998.89			1995.50		
-2LL	1965.33			1964.89			1953.50		

*N = 274, Obs = 752, measurement points: four (2 weeks before vacation, last workday, first day of work, 2 weeks after vacation)*.

*** *p < 0.001*;

** *p < 0.01*;

* *p < 0.05*.

### Self-Reported Work-Related Creativity Across Time and Recovery Experiences

With regard to recovery experiences predicting self-reported work-related creativity, the results of multilevel modeling indicated that, of the recovery experiences, detachment (*γ* = −0.13, *t* = −2.39, *p* = 0.02) predicted self-reported work-related creativity within persons, while mastery (*γ* = 0.25, *t* = 3.12, *p* = 0.002) was related to self-reported work-related creativity between persons (model 2, Table 4). Other recovery experiences were not related to self-reported work-related creativity. These findings suggest that at points in time during which employees experienced more difficulties detaching from work, they reported higher creativity, than at points in time at which they detached better from work. Moreover, employees who generally experienced more mastery experiences reported more creativity compared to employees experiencing lower levels of mastery. When, instead of including all six DRAMMA experiences in one model, the experiences are modeled one by one, the results show that detachment explains variance in self-reported work-related creativity within and between persons, while relaxation, mastery, autonomy, and affiliation explain variance between persons.

#### Additional Analysis

**Figure 2:**
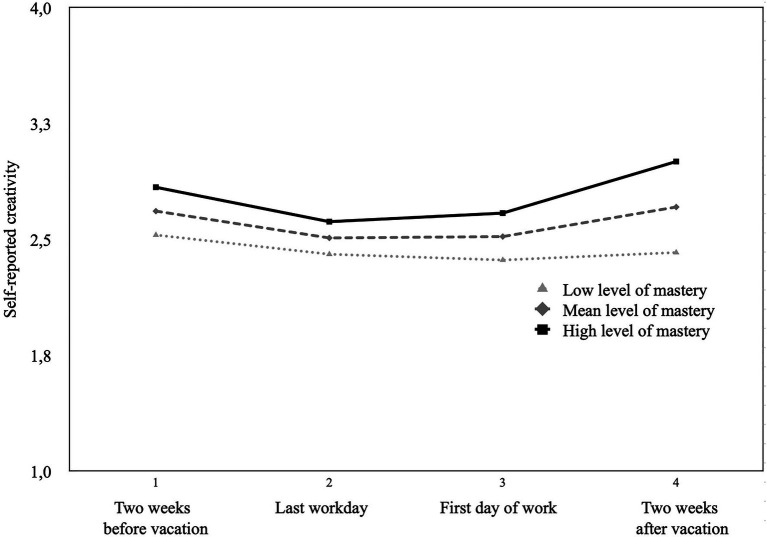
Self-reported work-related creativity on a five-point Likert scale over time for different levels of mastery experiences.

To examine the changes in creativity over time more closely, we analyzed whether the general level of a DRAMMA experience serves as a potential cross-level moderator. The results indicated that the general level of mastery experiences functions as a cross-level moderator (*γ* = 2.03, *t* = 2.04, *p* = 0.04). The results (model 3, Table 4; Figure 2) indicated that not only was employees’ general level of creativity higher if they experienced more mastery, but their increase in creativity after vacation was also steeper compared to that of employees with lower levels of mastery (simple slope analyses showed that the quadratic time slope was not significant for lower levels of mastery (*γ* = 0.08, *t* = 0.67, *p* = 0.50), while the slope was significant at medium (*γ* = 2.49, *t* = 3.27, *p* = 0.001) and enhanced levels of mastery experiences (*γ* = 4.19, *t* = 3.81, *p* < 0.001).

## Discussion

In this longitudinal study, we investigated changes in self-reported work-related creativity and examined how creativity changes after vacation compared to before vacation. Our analyses showed that employees subjectively perceived their creativity to be lower on the first day back at work after vacation. However, 2 weeks after vacation, employees also felt that they were more creative than before their holiday.

Why did employees perceive their creativity to be lower after vacation? These findings could be explained by workload after vacation. One might speculate as to whether on their first day back at work the employees were so immersed catching up with their work, trying to deal with tasks that accumulated during their absence (Kühnel and Sonnentag, 2011; Syrek et al., 2018), that they did not perceive themselves to be creative on this day. One could argue that creativity was perceived to be increased 2 weeks after their vacation because after 2 weeks back at work, the employees had successfully addressed the accumulated tasks and were able to focus on new tasks. Thus, they perceived their creativity as high and became aware of their enhanced capability to be creative.

Creativity at work is crucial for organizational success, particularly since the introduction and dissemination of digital and mobile information and communication technologies has significantly shaped the world of work. Recovery from work is key to restoring resources expended during work, and to maintaining health, well-being, mental productivity, and performance (Sonnentag and Geurts, 2009). Our study contributes to the literature as it provides evidence for intra- and interindividual relationships between recovery experiences and creativity. The results show that detachment was important for perceived creativity within persons, while mastery experiences were crucial in differences between persons. Specifically, the results indicated that employees perceived their creativity to be higher at times when they had more difficulties detaching from work. This finding may seem counter-intuitive as detachment from work can be regarded as an incubation phase which precedes creative thoughts (Sio and Ormerod, 2009; Ritter and Dijksterhuis, 2014). However, Vahle-Hinz et al. (2017) showed that problem-solving pondering was positively related to increases in creativity. Similarly, De Jonge et al. (2012) found that mental detachment was negatively related to creativity if employees had low problem-solving skills. Both studies examined differences between persons. Our study complements these findings by showing a similar pattern within persons, that is, that during times when people detach more, they are less creative than at times when they detach less. This illustrates that employees may get closer to a creative solution if they do not fully detach, but keep the work-related thoughts active on a low level. Spending more time thinking about work during leisure time could help to think outside of the box in order to make clever and complex connections (Vahle-Hinz et al., 2017).

Mastery could be identified as a key predictor of interindividual differences. Our results showed that employees with more mastery experiences reported more creativity than did other employees. Mastery experiences entail activities that encompass overcoming challenges and improving one’s skills (Newman et al., 2014). Intellectually stimulating tasks or physical challenges during leisure time may replenish employees’ emotional and cognitive resources. Moreover, positive affect evoked by mastery is highly conducive to creativity (Amabile et al., 2005).

### Strengths, Limitations, and Suggestions for Future Research

We conducted a longitudinal study with five measurement points, collected a large number of repeated observations, and examined self-reported work-related creativity. Despite this strong research design producing reliable evidence for relationships unfolding across time, our study suffers from shortcomings, on the basis of which we may offer recommendations for future research.

Even though higher creativity was observed 2 weeks after vacation, results from this non-experimental study cannot establish a causal effect of vacation. For causal inferences experimental designs were needed, for example, by randomly assigning participants to a vacation or a stay at home and work condition. However, random assignment of vacation is hard to achieve as family duties, school holidays etc. represent major practical challenges. Despite the longitudinal design we cannot rule out all alternative explanations and clearly establish causality as an experimental design would enable.

Further, observer-rated creative performance would have been an interesting addition to the self-reported measure. We had actually included the Alternative Uses task before and after vacation. However, due to a technical error, the two different tasks (i.e., generating creative used for a brick or a newspaper) were not counterbalanced correctly. Accordingly, this measure was not sound.

While the DRAMMA model does not specify interdependencies between the six experiences, experiences, such as autonomy, may also represent a moderator variable, influencing the strength of the relationship between the other DRAMMA experiences and creativity. Furthermore, it might be interesting for future research to examine temporal relationships between the DRAMMA experiences to analyze whether, for example, detachment is a necessary precondition and leads to mastery having a higher impact.

As all participants of the study had access to our vacation app “Holidaily,” which offers daily ideas on how to recover, we are not certain if the effects we found are attributable solely to the impact of vacation, or to what degree app usage played a role. Thus, recovery during vacation might have been enhanced compared to a vacation without a comparable app. However, we controlled for app usage, which did not influence the pattern of results.

The positive correlation between gender and creativity deserves to be mentioned (with females reporting higher levels of creativity than men). Earlier research on this relationship often showed no relationship between gender and self-reported creativity or findings consistent with common stereotypes on specific types of creativity and gender (e.g., males feeling more creative in science analytic and sports creativity and females in social communication and visual artistic creativity; Baer and Kaufman, 2008). The fact that we did find a significant relationship between creativity at work (mostly operationalized as having ideas to improve one’s work) and gender deserves closer attention in future research.

### Practical Implications

Our study is of value for employees and organizations who, in line with Staw (1995), argue that free time may be bad for morale and that there is no guarantee that creative ideas will emerge from vacation. We fully agree with Amabile that creativity does not come automatically. In our employees’ view, creativity is not increased immediately after vacation, but they perceived a surge in creativity 2 weeks after returning to work. While we cannot be certain if this association is caused by recovery, this finding is in line with the assumption that vacations could be a means to restore taxed resources from which employees can benefit in the following weeks as suggested by Sonnentag and Geurts (2009) and Newman et al. (2014).

Moreover, as mastery experiences were significantly related to creativity, it seems advisable to incorporate elements of challenge and learning opportunities in one’s vacation. If the results persist in an experimental study, one could derive that it rather seems that it is essential to learn how to think about work in a positive way (such as in problem-solving pondering), including when work-related thoughts become detrimental (Vahle-Hinz et al., 2017).

We set out to examine the link between vacations and creativity drawing on research from health psychology, organizational psychology, and leisure sciences. Our analyses provide evidence that (1) employees perceived their creativity to be lower immediately after their vacation but perceived an increase 2 weeks after being back at work, (2) employees’ detachment during leisure time was negatively associated with intraindividual differences in creativity, and (3) mastery experiences were positively related to interindividual differences in creativity.

## Data Availability Statement

The raw data supporting the conclusions of this article will be made available by the authors, without undue reservation.

## Ethics Statement

The studies involving human participants were reviewed and approved by Leuphana University of Lueneburg (reference number: 201606-EB-Antrag Lehr201606_holidaily). The patients/participants provided their written informed consent to participate in this study.

## Author Contributions

CS, JB, and DL contributed to conception and design of the study. CS performed the statistical analysis and wrote the first draft of the manuscript. JB wrote sections of the manuscript. All authors contributed to the article and approved the submitted version.

## Funding

This study was supported by the Academy of Finland (grant number: 308718). The data were collected as part of a larger data collection funded by the German health insurance company Barmer GEK. The funders did not influence in any way the analysis and presentation of the results or drafting of the manuscript.

## Conflict of Interest

The authors declare that the research was conducted in the absence of any commercial or financial relationships that could be construed as a potential conflict of interest.

## Publisher’s Note

All claims expressed in this article are solely those of the authors and do not necessarily represent those of their affiliated organizations, or those of the publisher, the editors and the reviewers. Any product that may be evaluated in this article, or claim that may be made by its manufacturer, is not guaranteed or endorsed by the publisher.

## Footnotes

^1^ https://osf.io/qj9ku/?view_only=94c84bf36cc2456a92c0443ad8dfc454

## References

[ref1] Achor S. Gielan M. (2016). The Data-Driven Case for Vacation. Available at: https://hbr.org/2016/07/the-data-driven-case-for-vacation (Accessed December 9, 2021).

[ref2] Amabile T. M. (1988). A model of creativity and innovation in organizations. Res. Organ. Behav. 10, 123–167.

[ref3] Amabile T. M. Barsade S. G. Mueller J. S. Staw B. M. (2005). Affect and creativity at work. Adm. Sci. Q. 50, 367–403. doi: 10.2189/asqu.2005.50.3.367, PMID: 34501671

[ref4] Amabile T. M. Conti R. Coon H. Lazenby J. Herron M. (1996). Assessing the work environment for creativity. Acad. Manag. J. 39, 1154–1184.

[ref5] Amelsvoort L. G. Kant I. Bültmann U. Swaen G. M. (2003). Need for recovery after work and the subsequent risk of cardiovascular disease in a working population. Occup. Environ. Med. 60, i83–i87. doi: 10.1136/oem.60.suppl_1.i83 12782752 PMC1765716

[ref6] Ashby F. G. Valentin V. V. Turken A. U. (2002). The effects of positive affect and arousal on working memory and executive attention. Adv. Conscious. Res. 44, 245–288. doi: 10.1075/aicr.44.11ash

[ref7] Baer J. Kaufman J. C. (2008). Gender differences in creativity. J. Creat. Behav. 42, 75–105. doi: 10.1002/j.2162-6057.2008.tb01289.x, PMID: 34707536

[ref8] Baumeister R. F. Leary M. R. (1995). The need to belong: desire for interpersonal attachments as a fundamental human motivation. Psychol. Bull. 117:497. doi: 10.1037/0033-2909.117.3.497, PMID: 7777651

[ref9] Binnewies C. Sonnentag S. Mojza E. J. (2009). Daily performance at work: feeling recovered in the morning as a predictor of day-level job performance. J. Organ. 30, 67–93. doi: 10.1002/job.541

[ref77] Binnewies C. Sonnentag S. Mojza E. J. (2010). Recovery during the weekend and fluctuations in weekly job performance: A week‐level study examining intra‐individual relationships. J. Occup. Organ. Psychol. 83, 419–441. doi: 10.1348/096317909X418049

[ref10] Bliese P. D. Ployhart R. E. (2002). Growth modeling using random coefficient models: model building, testing, and illustrations. Organ. Res. Methods 5, 362–387. doi: 10.1177/109442802237116

[ref11] Bosangit C. Hibbert S. McCabe S. (2015). If I was going to die I should at least be having fun. Ann. Tour. Res. 55, 1–14. doi: 10.1016/j.annals.2015.08.001

[ref12] Brajša-Žganec A. Merkaš M. Šverko I. (2011). Quality of life and leisure activities: how do leisure activities contribute to subjective well-being? Soc. Indic. Res. 102, 81–91. doi: 10.1007/s11205-010-9724-2, PMID: 34574775

[ref13] Callard F. Margulies D. S. (2014). What we talk about when we talk about the default mode network. Front. Hum. Neurosci. 8:619. doi: 10.3389/fnhum.2014.00619, PMID: 25202250 PMC4142807

[ref14] Chen Y. Lehto X. Y. Cai L. (2013). Vacation and well-being: a study of Chinese tourists. Ann. Tour. Res. 42, 284–310. doi: 10.1016/j.annals.2013.02.003

[ref15] Chen C. C. Petrick J. F. (2013). Health and wellness benefits of travel experiences: a literature review. J. Travel Res. 52, 709–719. doi: 10.1177/0047287513496477, PMID: 34435965

[ref16] Cheng C. Leung A. K. Wu T. Y. (2011). Going beyond the multicultural experience creativity link. J. Soc. Issues 67, 806–824. doi: 10.1111/j.1540-4560.2011.01729.x

[ref17] Cohen-Meitar R. Carmeli A. Waldman D. A. (2009). Linking meaningfulness in the workplace to employee creativity. Creat. Res. J. 21, 361–375. doi: 10.1080/10400410902969910

[ref22] De Bloom J. Geurts S. A. E. Kompier M. A. J. (2013). Vacation (after-) effects on employee health and well-being, and the role of vacation activities, experiences and sleep. J. Happiness Stud. 14, 613–633. doi: 10.1007/s10902-012-9345-3

[ref18] De Bloom J. Kompier M. Geurts S. De Weerth C. Taris T. Sonnentag S. (2009). Do we recover from vacation? Meta-analysis of vacation effects on health and well-being. J. Occup. Health 51, 13–25. doi: 10.1539/joh.K8004, PMID: 19096200

[ref19] de Bloom J. Nawijn J. Geurts S. Kinnunen U. Korpela K. (2017). Holiday travel, staycations, and subjective well-being. J. Sustain. Tour. 25, 573–588. doi: 10.1080/09669582.2016.1229323

[ref20] De Bloom J. Radstaak M. Geurts S. (2014a). Vacation effects on behaviour, cognition and emotions of compulsive and non-compulsive workers. Stress. Health 30, 232–243. doi: 10.1002/smi.2600 25100274

[ref21] De Bloom J. Ritter S. Kühnel J. Reinders J. Geurts S. (2014b). Vacation from work: a ‘ticket to creativity’? The effects of recreational travel on cognitive flexibility and originality. Tour. Manag. 44, 164–171. doi: 10.1016/j.tourman.2014.03.013

[ref23] De Jonge J. Spoor E. Sonnentag S. Dormann C. van den Tooren M. (2012). “Take a break?!” off-job recovery, job demands, and job resources as predictors of health, active learning, and creativity. Eur. J. Work Organ. Psychol. 21, 321–348. doi: 10.1080/1359432X.2011.576009

[ref24] Deci E. L. Ryan R. M. (1987). The support of autonomy and the control of behavior. J. Pers. Soc. Psychol. 53, 1024–1037. doi: 10.1037/0022-3514.53.6.1024, PMID: 3320334

[ref25] Deci E. L. Ryan R. M. (2000). The “what” and “why” of goal pursuits: human needs and the self-determination of behavior. Psychol. Inq. 11, 227–268. doi: 10.1207/S15327965PLI1104_01, PMID: 20204932

[ref26] Eaker E. D. Pinsky J. Castelli W. P. (1992). Myocardial infarction and coronary death among women: psychosocial predictors from a 20-year follow-up of women in the Framingham study. Am. J. Epidemiol. 135, 854–864. doi: 10.1093/oxfordjournals.aje.a116381, PMID: 1585898

[ref27] Eden D. (2001). “Vacations and other respites: studying stress on and off the job,” in Well-Being in Organizations. eds. C. Cooper and I. T. Robertson (West Sussex, UK: John Wiley & Sons, Ltd), 305–330.

[ref28] Elsbach K. D. Hargadon A. B. (2006). Enhancing creativity through “mindless” work. Organ. Sci. 17, 470–483. doi: 10.1287/orsc.1060.0193

[ref29] Filep S. (2012). “Positive psychology and tourism,” in Handbook of Tourism and Quality-of-Life Research. eds. Uysal M. Perdue M. Sirgy J. (Dordrecht: Springer), 31–50.

[ref30] Fink A. Grabner R. H. Gebauer D. Reishofer G. Koschutnig K. Ebner F. (2010). Enhancing creativity by means of cognitive stimulation: evidence from an fMRI study. NeuroImage 52, 1687–1695. doi: 10.1016/j.neuroimage.2010.05.072, PMID: 20561898

[ref31] Fredrickson B. L. (2002). “Positive emotions,” in Handbook of Positive Psychology. eds. Snyder C. R. Lopez S. J. (New York: Oxford University Press), 120–134.

[ref32] Fritz C. Sonnentag S. (2006). Recovery, well-being, and performance-related outcomes: the role of workload and vacation experiences. J. Appl. Psychol. 91, 936–945. doi: 10.1037/0021-9010.91.4.936, PMID: 16834516

[ref33] George J. M. Zhou J. (2001). When openness to experience and conscientiousness are related to creative behavior. J. Appl. Psychol. 86:513. doi: 10.1037/0021-9010.86.3.513, PMID: 11419810

[ref34] Gilbert D. Abdullah J. (2004). Holiday taking and the sense of wellbeing. Ann. Tour. Res. 31, 103–121. doi: 10.1016/j.annals.2003.06.001

[ref35] Goldsmith R. E. Matherly T. A. (1988). Creativity and self-esteem: a multiple operationalization validity study. J. Psychol. 122, 47–56. doi: 10.1080/00223980.1988.10542942

[ref78] Grabner R. H. Fink A. Neubauer A. C. (2007). Brain correlates of self-rated originality of ideas: Evidence from event-related power and phase-locking changes in the EEG. Behav. Neurosci. 121, 224–230. doi: 10.1037/0735-7044.121.1.224 17324067

[ref36] Greenberg E. (1992). Creativity, autonomy, and evaluation of creative work: artistic workers in organizations. J. Creat. Behav. 26, 75–80. doi: 10.1002/j.2162-6057.1992.tb01162.x

[ref38] Gump B. B. Matthews K. A. (2000). Are vacations good for your health? The 9-year mortality experience after the multiple risk factor intervention trial. Psychosom. Med. 62, 608–612. doi: 10.1097/00006842-200009000-00003, PMID: 11020089

[ref39] Hackman J. R. Oldham G. R. (1974). The Job Diagnostic Survey: An Instrument for the Diagnosis of Jobs and the Evaluation of Job Redesign Projects. New Haven: Yale University.

[ref40] Hammond M. M. Neff N. L. Farr J. L. Schwall A. R. Zhao X. (2011). Predictors of individual-level innovation at work: a meta-analysis. Psychol. Aesthet. Creat. Arts 5:90. doi: 10.1037/a0018556, PMID: 19702361

[ref41] Hunter-Jones P. (2004). Young people, holiday taking and cancer: an exploratory analysis. Tour. Manag. 25, 249–258. doi: 10.1016/S0261-5177(03)00094-3

[ref42] Johnston M. M. Finney S. J. (2010). Measuring basic needs satisfaction. Contemp. Educ. Psychol. 35, 280–296. doi: 10.1016/j.cedpsych.2010.04.003, PMID: 34733198

[ref43] Koo T. K. Li M. Y. (2016). A guideline of selecting and reporting intraclass correlation coefficients for reliability research. J. Chiropr. Med. 15, 155–163. doi: 10.1016/j.jcm.2016.02.012, PMID: 27330520 PMC4913118

[ref44] Kühn S. Ritter S. M. Müller B. C. Van Baaren R. B. Brass M. Dijksterhuis A. (2014). The importance of the default mode network in creativity: a structural MRI study. J. Creat. Behav. 48, 152–163. doi: 10.1002/jocb.45

[ref45] Kühnel J. Sonnentag S. (2011). How long do you benefit from vacation? A closer look at the fade-out of vacation effects. J. Organ. Behav. 32, 125–143. doi: 10.1002/job.699

[ref46] Kujanpää M. Syrek C. Lehr D. Kinnunen U. Reins J. A. de Bloom J. (2020). Need satisfaction and optimal functioning at leisure and work: a longitudinal validation study of the DRAMMA model. J. Happiness Stud. 22, 681–707. doi: 10.1007/s10902-020-00247-3

[ref47] Lehto X. Y. Choi S. Lin Y.-C. MacDermid S. M. (2009). Vacation and family functioning. Ann. Tour. Res. 36, 459–479. doi: 10.1016/j.annals.2009.04.003, PMID: 29480917

[ref49] McCabe S. Johnson S. (2013). The happiness factor in tourism: subjective well-being and social tourism. Ann. Tour. Res. 41, 42–65. doi: 10.1016/j.annals.2012.12.001

[ref50] McLachlan S. Hagger M. S. (2010). Effects of an autonomy-supportive intervention on tutor behaviours in a higher education context. Teach. Teach. Educ. 26, 1204–1210. doi: 10.1016/j.tate.2010.01.006

[ref51] Miyakawa E. Kawakubo A. Oguchi T. (2019). Do people who travel more perform better at work? Int. J. Tour. Res. 21, 427–436. doi: 10.1002/jtr.2269, PMID: 31934845

[ref52] Mohr G. Müller A. Rigotti T. Aycan Z. Tschan F. (2006). The assessment of psychological strain in work contexts. Eur. J. Psychol. Assess. 22, 198–206. doi: 10.1027/1015-5759.22.3.198, PMID: 31229529

[ref53] Newman D. B. Tay L. Diener E. (2014). Leisure and subjective well-being: a model of psychological mechanisms as mediating factors. J. Happiness Stud. 15, 555–578. doi: 10.1007/s10902-013-9435-x

[ref54] Noy C. (2004). This trip really changed me: backpackers’ narratives of self-change. Ann. Tour. Res. 31, 78–102. doi: 10.1016/j.annals.2003.08.004

[ref55] Raudenbush S. W. Bryk A. S. (2002). Hierarchical Linear Models: Applications and Data Analysis Methods. Vol. 1. London: Sage.

[ref56] Ritter S. Damian R. Simonton D. K. Baaren R. Strick M. Derks J.. (2012). Diversifying experiences enhance cognitive flexibility. J. Exp. Soc. Psychol. 48, 961–964. doi: 10.1016/j.jesp.2012.02.009

[ref57] Ritter S. M. Dijksterhuis A. (2014). Creativity—the unconscious foundations of the incubation period. Front. Hum. Neurosci. 8:215. doi: 10.3389/fnhum.2014.00215, PMID: 24782742 PMC3990058

[ref58] Schon D. A. (1983). The Reflective Practitioner. New York: Basic Books.

[ref59] Shaw S. M. Havitz M. E. Delemere F. M. (2008). I decided to invest in my kids' memories: family vacations, memories, and the social construction of the family. Tour. Cult. Commun. 8, 13–26. doi: 10.3727/109830408783900361

[ref62] Sio U. N. Ormerod T. C. (2009). Does incubation enhance problem solving? A meta-analytic review. Psychol. Bull. 135:94. doi: 10.1037/a0014212, PMID: 19210055

[ref63] Smith S. M. Blankenship S. E. (1989). Incubation effects. Bull. Psychon. Soc. 27, 311–314. doi: 10.3758/BF03334612, PMID: 34844202

[ref64] Sonnentag S. Fritz C. (2007). The recovery experience questionnaire: development and validation of a measure for assessing recuperation and unwinding from work. J. Occup. Health Psychol. 12:204. doi: 10.1037/1076-8998.12.3.204, PMID: 17638488

[ref65] Sonnentag S. Geurts S. A. (2009). “Methodological issues in recovery research,” in Current Perspectives on Job-Stress Recovery. eds. Sonnentag S. Perrewé P. L. Ganster D. C. (Emerald Group Publishing Limited), 1–36.

[ref66] Sonnentag S. Venz L. Casper A. (2017). Advances in recovery research: what have we learned? What should be done next? J. Occup. Health Psychol. 22:365. doi: 10.1037/ocp0000079, PMID: 28358572

[ref67] Spreitzer G. Sutcliffe K. Dutton J. Sonenshein S. Grant A. M. (2005). A socially embedded model of thriving at work. Organ. Sci. 16, 537–549. doi: 10.1287/orsc.1050.0153, PMID: 34421715

[ref68] Staw B. (1995). “Why no one really wants creativity,” in Creative Action in Organizations: Ivory Tower Visions & Real World Voices. eds. Ford C. Gioia D. (Thousand Oaks, CA: SAGE Publications, Inc.), 161–166.

[ref69] Stebbins R. A. (2005). Choice and experiential definitions of leisure. Leis. Sci. 27, 349–352. doi: 10.1080/01490400590962470

[ref70] Stephens J. P. Carmeli A. (2017). “Relational leadership and creativity: the effects of respectful engagement and caring on meaningfulness and creative work involvement,” in Handbook of Research on Leadership and Creativity. eds. Mumford M. D. Hemlin S. (Edward Elgar Publishing).

[ref71] Syrek C. J. Weigelt O. Kühnel J. de Bloom J. (2018). All I want for Christmas is recovery–changes in employee affective well-being before and after vacation. Work Stress. 32, 313–333. doi: 10.1080/02678373.2018.1427816

[ref72] Taylor C. W. (1988). “Various approaches to and definitions of creativity,” in The Nature of Creativity. ed. Sternberg R. J. (Cambridge: Cambridge University Press), 99–124.

[ref73] Vahle-Hinz T. Mauno S. de Bloom J. Kinnunen U. (2017). Rumination for innovation? Analysing the longitudinal effects of work-related rumination on creativity at work and off-job recovery. Work Stress. 31, 315–337. doi: 10.1080/02678373.2017.1303761

[ref74] Volmer J. Richter S. Syrek C. J. (2019). Creative at each age: age-related differences in drivers of workplace creativity from an experience sampling study. J. Creat. Behav. 53, 531–545. doi: 10.1002/jocb.233

[ref75] Westman M. Etzion D. (2001). The impact of vacation and job stress on burnout and absenteeism. Psychol. Health 16, 595–606. doi: 10.1080/08870440108405529, PMID: 22804501

[ref76] Yau C. (1991). An essential interrelationship: healthy self-esteem and productive creativity. J. Creat. Behav. 25, 154–161. doi: 10.1002/j.2162-6057.1991.tb01365.x

