# Supplementary material for: Well Recovered and More Creative? A Longitudinal Study on the Relationship Between Vacation and Creativity
Source: Front Psychol. 2021 Dec 23;12:784844. doi: 10.3389/fpsyg.2021.784844 (PMC8733151; doi:10.3389/fpsyg.2021.784844)
Supplement: Supplementary file 1 [file Data_Sheet_1.PDF]

Supplemental material - Measures

Well recovered and more creative? A longitudinal study on the relationship between vacation and creativity

Christine J. Syrek, Jessica de Bloom, Dirk Lehr

3.4. Measures

Self-reported work-related creativity was assessed at four measurement points: two weeks prior to the vacation, on the last day at work, on the first day back at work, and two weeks after the vacation with three items adapted from George and Zhou (2001).

"My head is full of creative and innovative ideas that are related to my work."

"My head was full of creative solutions to work-related problems."

"My head was full of ideas to solve work tasks in a new way."

Participants responded on a five-point Likert scale (1 = totally disagree, 5 = totally agree). Mean Cronbach's alpha was .90.

Recovery experiences were assessed at five measurement points: two weeks prior to vacation, on the last day at work, during the vacation, and on the first day back at work, as well as two weeks after returning from vacation.

The questionnaire used to assess recovery experiences builds on and combines existing items to cover all dimensions of the DRAMMA model, referring to the participant's leisure time. Participants responded on a five-point Likert scale (1 = totally disagree, 5 = totally agree). The DRAMMA model has been validated with regard to vacation experiences and well-being by Kujanpää et al. (2020).

Detachment is measured with three items adapted from the well-validated recovery experience questionnaire (Sonnentag & Fritz, 2007) and the rumination scale (Mohr, Müller, Rigotti, Aycan, & Tschan, 2006).

"I forgot about unpleasant duties and work tasks."

"I distanced myself from unpleasant duties and work tasks."

"I gained distance from the demands of work."

Relaxation is measured using the well-validated recovery experience questionnaire with three items each (Sonnentag & Fritz, 2007).

"I kicked back and relaxed."

"I did relaxing things."

“I used the time to relax.”

Mastery is measured using the well-validated recovery experience questionnaire with three items each (Sonnentag & Fritz, 2007).

„I learned new things.“

“I did things that challenge me.”

“I did something to broaden my horizon.”

To measure *meaning*, three items from the “job diagnostic survey” (Hackman & Oldham, 1974) were reformulated to apply to leisure time.

“I did something that was important to me.”

“I have done something meaningful.”

“I did something that was important to me personally.”

Autonomy is measured with three items adapted from the “Basic Need Satisfaction in General Scale” (Johnston & Finney, 2010).

“I felt like I am free to decide for myself how to spend my time.”

“I felt free to express my ideas and opinions.”

“I felt free to do what I want.”

Affiliation is measured with three items adapted from the “Basic Need Satisfaction in General Scale” (Johnston & Finney, 2010).

„I liked the people I interacted with.”

“I felt close to the people I was interacting with.”

“The people I interacted with paid attention how I feel.”

Mean Cronbach’s alpha was .94 for detachment, .95 for relaxation, .83 for autonomy, .94 for mastery, .92 for meaning and .93 for affiliation.
