# Supplementary material for: Well Recovered and More Creative? A Longitudinal Study on the Relationship Between Vacation and Creativity
Source: Front Psychol. 2021 Dec 23;12:784844. doi: 10.3389/fpsyg.2021.784844 (PMC8733151; doi:10.3389/fpsyg.2021.784844)
Supplement: Supplementary file 2 [file Data_Sheet_2.PDF]

Supplemental material – Mean scores

Well recovered and more creative? A longitudinal study on the relationship between vacation and creativity

Christine J. Syrek, Jessica de Bloom, Dirk Lehr

Descriptive statistics for recovery experiences and creativity

Table 5. Mean scores for recovery experiences and self-reported work-related creativity across all time points.

	Two weeks before vacation	Last workday	During vacation	First workday	Two weeks after vacation
Detachment	2.71	2.45	3.93	3.97	3.28
Relaxation	2.55	2.36	3.83	3.90	3.26
Autonomy	3.08	2.91	3.99	4.00	3.53
Mastery	2.84	2.79	3.32	3.43	3.18
Meaning	3.02	2.99	3.55	3.64	3.45
Affiliation	3.91	3.74	4.39	4.33	4.08
Creativity	2.67	2.58		2.48	2.80

Note. $N = 274$.

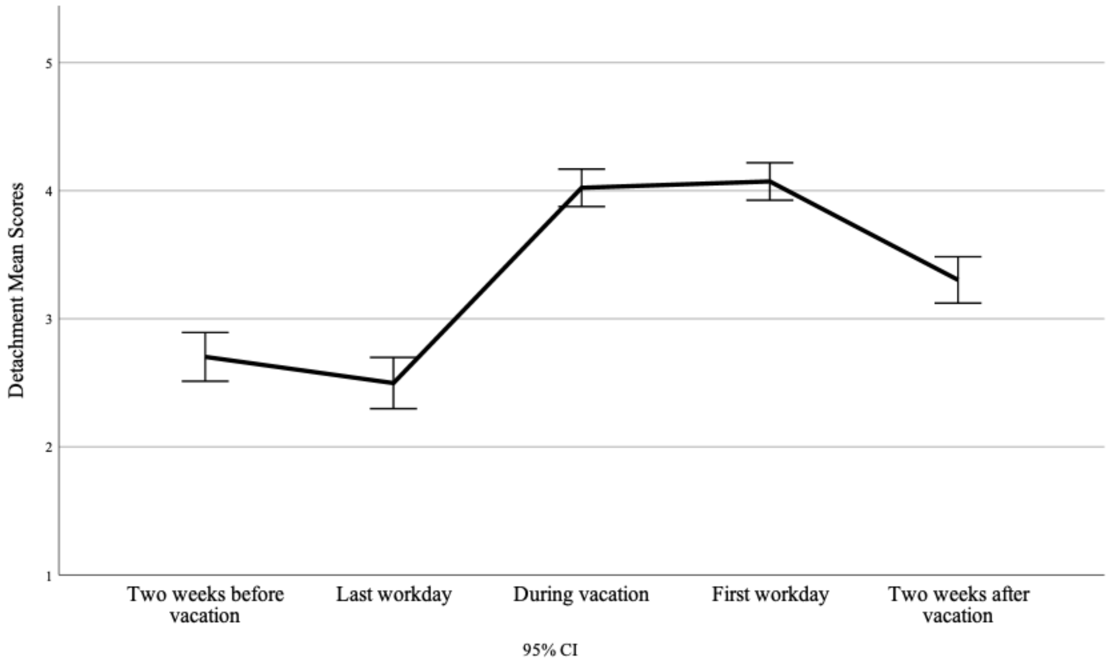

Figure 3. Mean scores of detachment across time on a 5-point Likert scale.

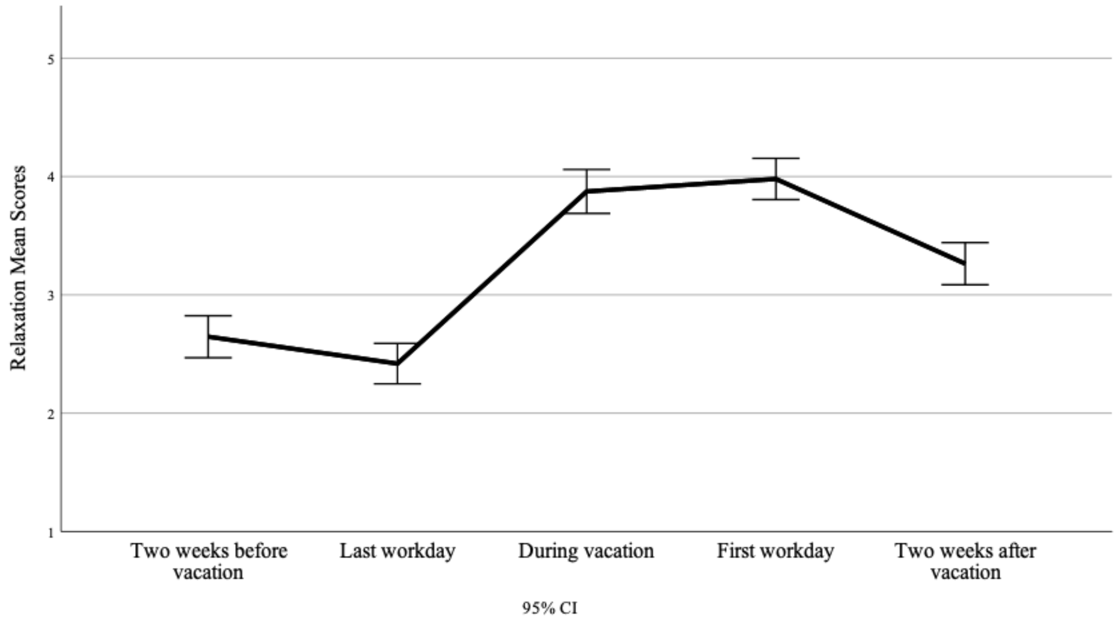

Figure 4. Mean scores of relaxation across time on a 5-point Likert scale.

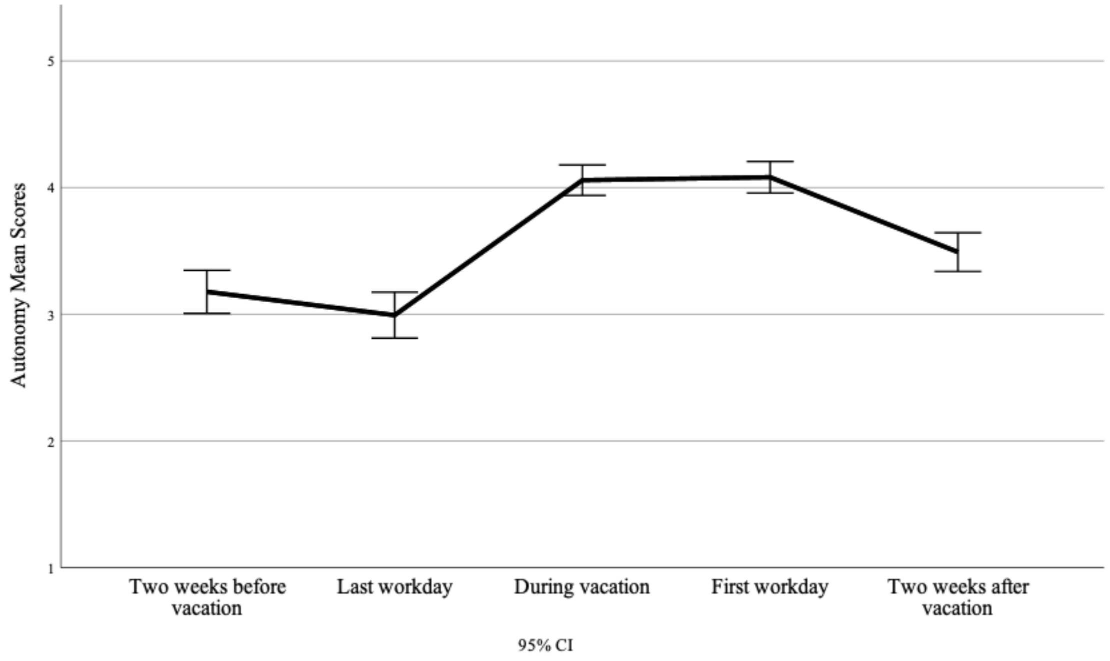

Figure 5. Mean scores of autonomy across time on a 5-point Likert scale.

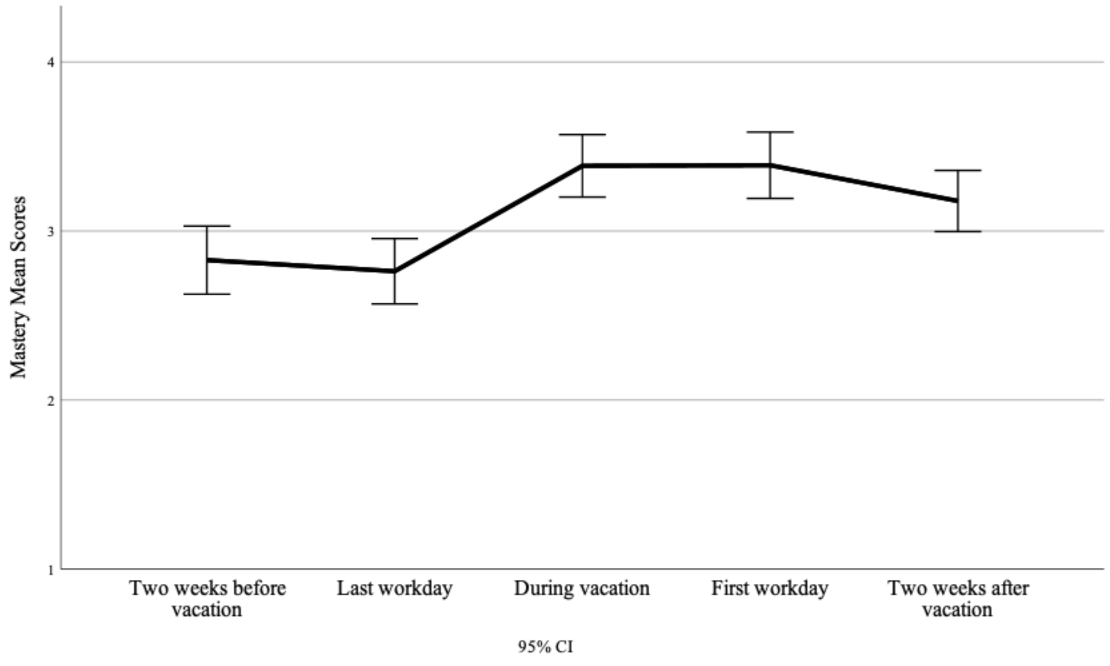

Figure 6. Mean scores of mastery across time on a 5-point Likert scale.

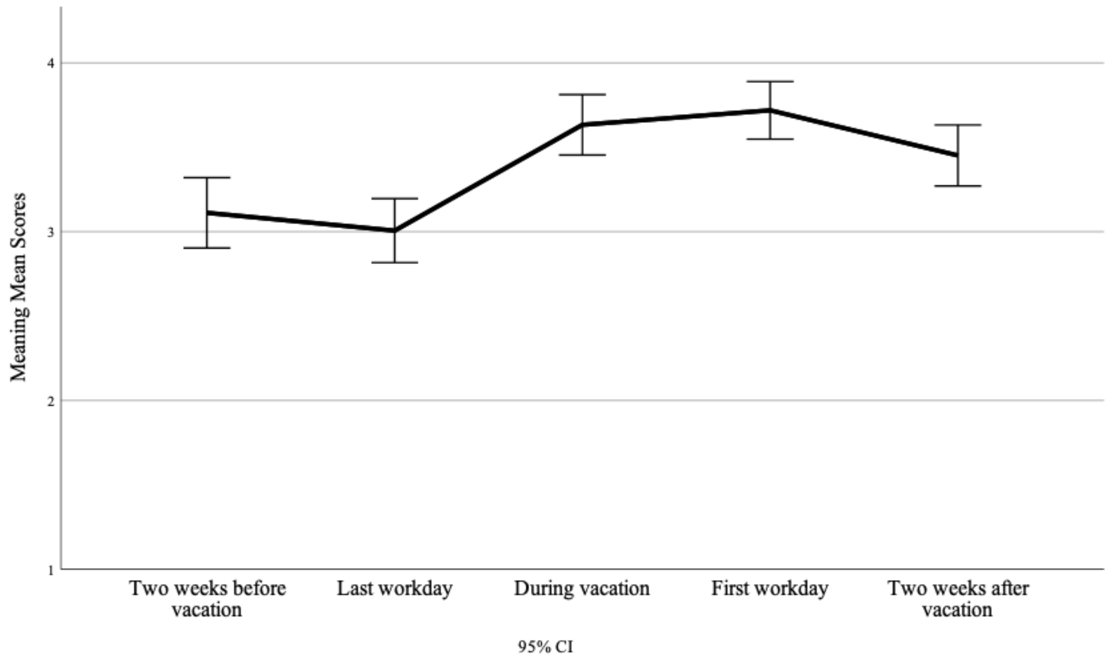

Figure 6. Mean scores of meaning across time on a 5-point Likert scale.

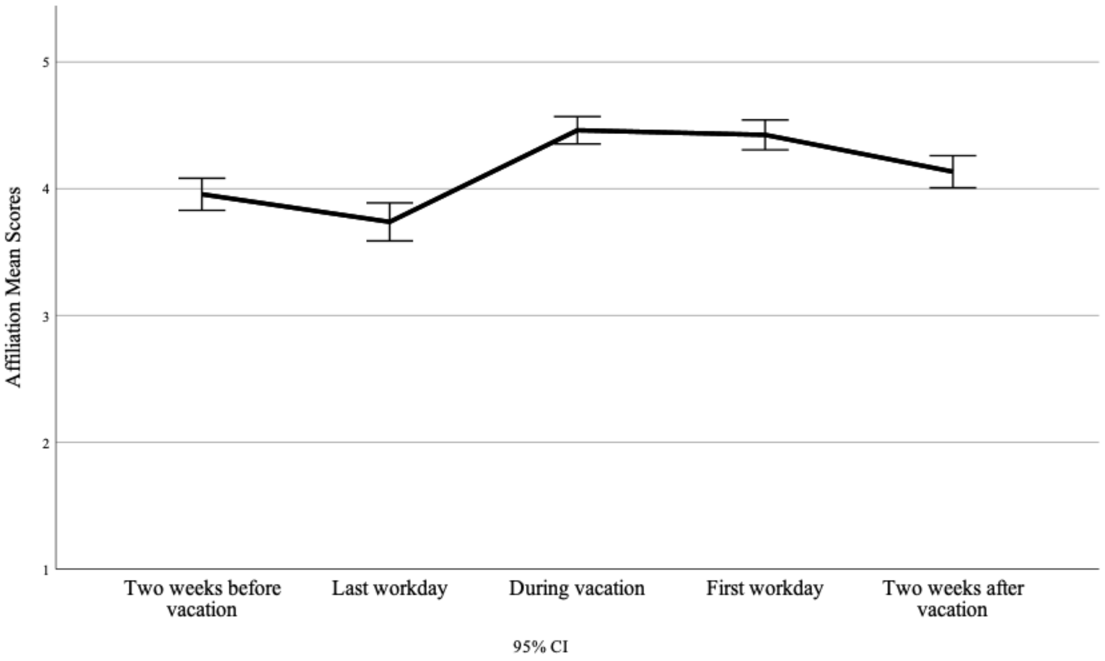

Figure 7. Mean scores of affiliation across time on a 5-point Likert scale.

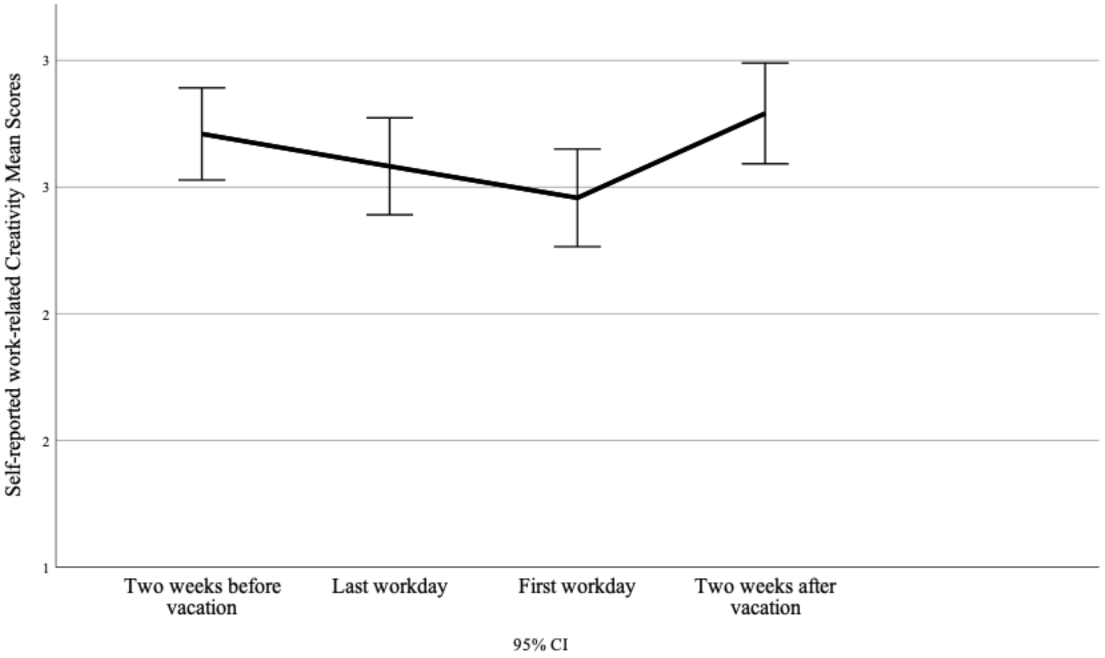

Figure 8. Mean scores of self-reported work-related creativity across time on a 5-point Likert scale.
